# The Influence of Plantar Short Foot Muscle Exercises on Foot Posture and Fundamental Movement Patterns in Long-Distance Runners, a Non-Randomized, Non-Blinded Clinical Trial

**DOI:** 10.1371/journal.pone.0157917

**Published:** 2016-06-23

**Authors:** Iwona Sulowska, Łukasz Oleksy, Anna Mika, Dorota Bylina, Jarosław Sołtan

**Affiliations:** 1 Department of Clinical Rehabilitation, University of Physical Education in Krakow, Krakow, Poland; 2 Zen Machines Poland, Łańcut, Poland; 3 Department of Biology and Anatomy, The Josef Pilsudski University of Physical Education in Warsaw, Faculty of Physical Education in Biala Podlaska, Biala Podlaska, Poland; 4 Foreign Languages Department, The Josef Pilsudski University of Physical Education in Warsaw, Faculty of Physical Education in Biala Podlaska, Biala Podlaska, Poland; Vanderbilt University, UNITED STATES

## Abstract

**Background:**

The objective of this study was **t**o evaluate the influence of two kinds of plantar short foot muscles exercise on foot posture and fundamental movement patterns in long-distance runners.

**Design:**

A parallel group non-blinded trial with 6-week follow-up.

**Methods:**

Twenty five long-distance runners aged 22–35 years. They were divided into two groups. In group 1 (n = 13) subjects performed the exercise “Vele’s Forward Lean” and “Reverse Tandem Gait” and in Group 2 (n = 12) the “Short Foot Exercise.” The runners performed the exercises daily for 6 weeks. The Foot Posture Index (FPI-6) and The Functional Movement Screen (FMS) tests were performed twice: at baseline and after 6 weeks of the exercise.

**Results:**

A significant improvement was observed in FPI -6 (talar head palpation in Group 1, and inversion/eversion of the calcaneus in Group 2). Also in Group 1 a significant improvement was noted in FMS tests: deep squat, active straight leg raise and in total score.

**Conclusions:**

Short foot muscles strengthening exercises have beneficial effect on functional movement patterns and on foot posture, therefore they should be included as a part of daily training program of runners.

**Trial Registration:**

Australian New Zealand Clinical Trials Registry ACTRN12615001200572

## Introduction

The plantar intrinsic foot muscles play a crucial role in supporting the medial longitudinal arch, providing the foot stability and flexibility for shock absorption. These muscles also have an influence on the range of foot pronation [1]. Kernozek et al. [2] have reported that individuals with flat-arched foot have significantly higher pronation in stance than high-arched individuals. There are some studies that describe the relationship between excessive foot pronation and increased risk of acute injury or repetitive strain injury [3,4]. Researchers have assessed the changes in foot posture after a long-distance run [5,6]. When the plantar intrinsic foot muscles were fatigued, there was a change in foot posture towards pronated position [5–7]. Excessive pronation transmitted to internal rotation of the tibia, may cause overloading of the knee joint or may be the cause of other changes in proximal part of the lower extremity [1,8].

According to Myers [9] the anatomical and functional connection between the foot and the upper part of the body is the superficial back line. It contains the plantar fascia and short toe flexors, the achilles and the triceps surae, the hamstrings, the sacrotuberous ligament, the fascia of the sacrolumbar area, the erector spinae and the epicranial fascia [9]. Previous studies have also indicated the presence of continuity and connectivity between fascia or muscle that may be anatomically distant from each other [10]. Moreover anatomical dissections have confirmed the continuity of the fascial system in the upper and the lower limbs [11]. Due to a significant role of the plantar intrinsic foot muscles, not only in relation to the foot, but also indirectly to the whole biokinematic chain, correct and optimal training of these muscles seems very significant. This is especially important for long-distance runners as they are exposed to repetitive loads [1].

Insufficient foot muscles strength may be the reason of excessive foot loading during run and the tension may be transmitted to upper parts of the superficial back line leading to overload and functional restrictions [4,9,12]. It has been also suggested that any tension at a particular part of this system may have detrimental effects resulting in global decreased flexibility [9]. For example, reduced flexibility and tightness in the hamstrings [9,13] and tightness in the calf muscles are a possible etiological factor for plantar fasciitis [14,15].

To date the majority of studies investigating short foot muscles exercises have focused on their influence on rehabilitation after injuries of the ankle, on pes planus, on falls prevention in older people, on balance parameters, and the medial longitudinal arch morphology [15–19].

There is a lack of studies examining the effects of plantar short foot muscles exercises on long-distance runners. Therefore this study, for the first time, undertakes this subject. Accordingly, in this study we sought to examine the influence of two kinds of plantar short foot muscles exercises performed by long-distance runners daily for 6 weeks. This work is particularly novel as it takes a comprehensive approach by examining the effects of short foot exercises on fundamental movement patterns and foot posture in long distance runners.

## Materials and Methods

### Participants

Twenty five long distance runners (11 females and 14 males) aged 22–35 years (mean±Standard Deviation (SD) 28±3.86), who run regularly 3–7 times a week with a total distance of 30–100 km per week (mean±SD 59.2±22.11 km) participated in this study. The running distance was unchanged during the study and all the runners were instructed not to change their training routine in any way in order not to affect the results. Runners were excluded if they had a previous history of acute injury up to six months prior to the enrollment on the study. The recruitment and follow-up of the study participants was performed from April to July 2015. All measurements were performed by one examiner who was blinded to the group allocation. All the subjects were informed in detail about the research protocol and gave their written informed consent to participate in the study. The approval of the Ethical Committee of Regional Medical Chamber in Krakow had been obtained before the study. This study was registered in the Australian New Zealand Clinical Trials Registry (ANZCTR). Registration number: ACTRN12615001200572.

The trial was registered retrospectively because it did not include any drug or medical intervention. The kind of intervention (exercise) allows us to register the trial as ongoing study after the first participant enrollment. The data presented in the current study are a part of a wider project. The authors confirm that all ongoing and related trials for this intervention are registered.

### Procedures

**Fig 1:**
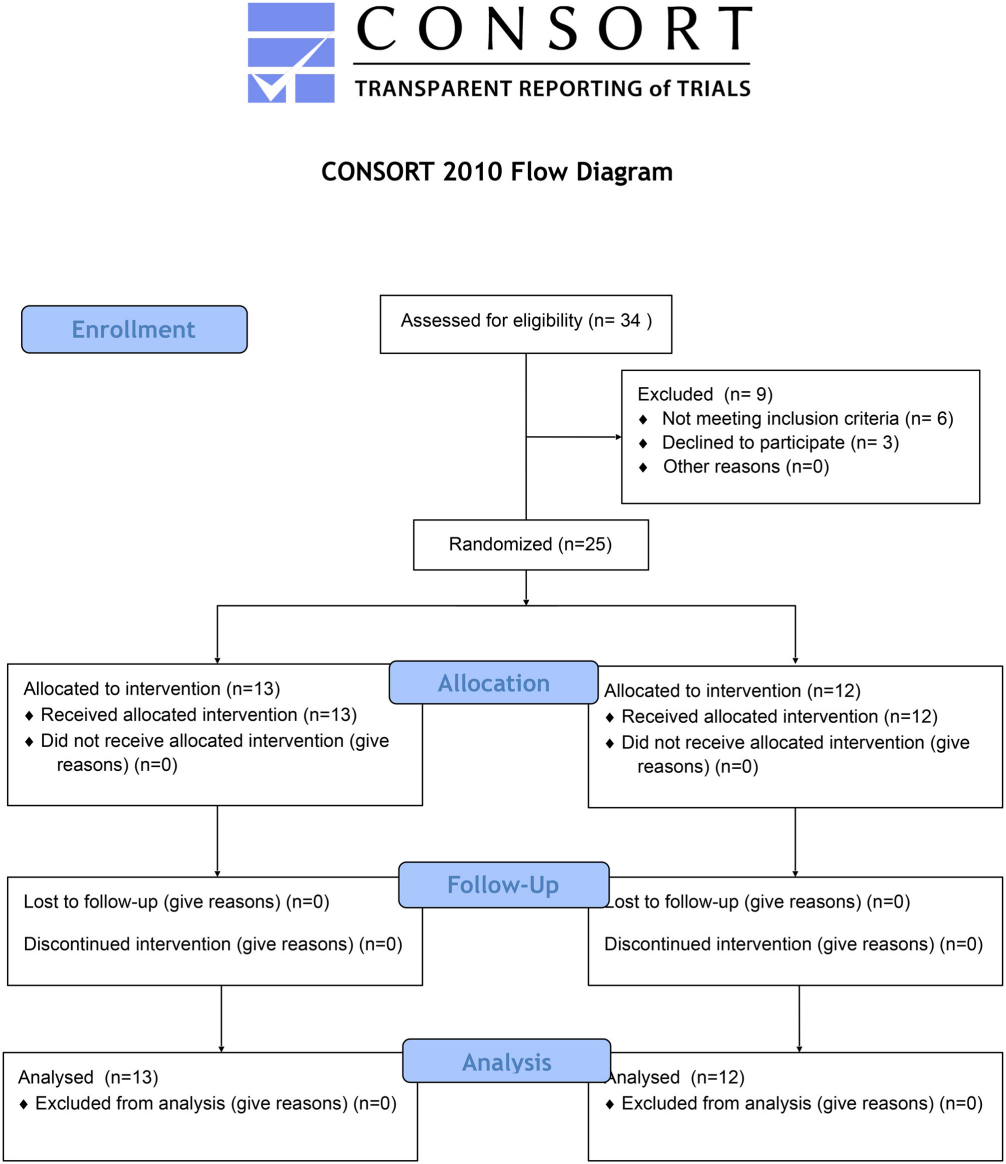
Consort diagram.

All study participants were divided into two groups: Group 1 (n = 13) in which subjects performed the “Vele’s Forward Lean” and “Reverse Tandem Gait” exercises and Group 2 (n = 12) in which subjects performed the “Short Foot Exercise.” (Fig 1). The two groups were not randomized. All runners in both groups have performed the exercises daily for 6 weeks. All measurements have been performed twice: at baseline and after 6 weeks of the exercising.

The Foot Posture Index (FPI-6) [20] is used for multidimensional and comprehensive evaluation of the feet. Due to its non-specific character, it has a wide clinical application in assessment of the risk of injury in athletes [21]. The intra-rater reliability of the FPI-6 between two sessions reported by Terada et al. [22] was ICC_3.3_ = 0.95; 95%CI. FPI-6 is composed of six parts that evaluate particular components of the front foot and the rear foot:

1. Talar head palpation,
2. Supra and infra lateral malleolar curvature,
3. Inversion/eversion of the calcaneus,
4. Prominence in the region of the talonavicular joint,
5. Height and congruence of the medial longitudinal arch,
6. Abduction/adduction of the forefoot on the rear foot.

Each of these parts is evaluated on a scale from -2 to +2. The negative values indicate supination, while the positive values indicate pronation. The neutral position of the foot is categorized as 0. The total FPI-6 result allows to classify the foot into the following categories:

-12 to-5: increased supination,
-4 to-1: slight supination,
0 to+5: neutral foot,
+6 to+9: slight pronation,
+10 to+12: increased pronation.

The Functional Movement Screen (FMS) test [23] is a comprehensive evaluation tool which estimates the quality of the fundamental movement patterns that require a balance of stability, mobility, strength, coordination and normal neuromuscular control. As was reported that intra-class correlation coefficients for intra-rater reliability of FMS ranged 0.81–0.91 [24]. The FMS test comprise seven movement patterns:

1. Deep squat,
2. Hurdle step,
3. In-line lunge,
4. Shoulder mobility,
5. Active straight leg raise,
6. Trunk stability push up,
7. Rotary stability.

Each of the above movement patterns is evaluated on a scale from 0 to 3. During the tests, each runner was observed from the front, the back and the side in frontal and sagittal plane. In asymmetric tests both left and right sides were assessed. The lower score was counted toward the total. Each test was performed three times. The maximum score possible to achieve was 21.

The runners were divided into two groups. All subjects were fully familiarized with the exercise protocol and were additionally given a written instruction copy. They were recommended to perform the plantar short foot muscles exercises daily, twice a day for 15 minutes each time during six weeks. The exercise based on “short foot” are a part of Sensory Motor Stimulation Method described by Janda [25]. Short foot is a posture of the foot in which the medial and longitudinal arches are raised to improve the foot’s biomechanical position and to activate the intrinsic muscles of the feet in a tonic manner. It forces the foot to be placed in a more neutral and less-pronated position, improves the stability of the body in the upright position, and helps to improve the required springing moment of the foot during walking. The exercises were performed barefoot with the proper loading of three support points of the feet, the first and the fifth metatarsal heads and the heel. The correctness of the exercise performance was monitored by examiner once a week during a meeting before training.

Group 1 (n = 13) performed the exercises: “Vele’s Forward Lean” and “Reverse Tandem Gait”. The first exercise constituted of the maximal forward lean from a standing position with arms alongside the body and with feet shoulder-width apart, with body in line, without lifting heels off the floor. The “Reverse Tandem Gait” exercise constituted of walking backwards. During forward lean and during walking backwards the short foot position was maintained according to Janda’s Sensory Motor Stimulation Method. First, the metatarsus were loaded, then the heel [25,26].

Group 2 (n = 12) performed the “Short Foot Exercise”. The exercise consisted of two stages: the first shortening the foot in the anterior-posterior direction and attempting to bring the head of the metatarsals towards the heel without toe flexion, and—the second—balanced loading of the three support points of the foot. The toes should be relaxed whereas the forefoot and heel should be kept on the ground. The exercises comprised three variations and lasted for 15 minutes altogether, where each variation possessed gradually increased level of difficulty: the sitting position, the standing position and the half-squat [25,26]. The runners added each consecutive variation of the exercise to their routine as soon as they mastered the previous one. Thus at the beginning the runners performed the exercise in the sitting position for 15 minutes, after the sitting position was mastered, the runners exercises for 7,5 minutes in the sitting position and for 7,5 minutes in the standing position. Accordingly, after the standing position was also mastered the runners performed the exercise for 5 minutes in the sitting position, 5 minutes in the standing position and 5 minutes in the half-squat, till the end of the experiment.

### Statistical analysis

The statistical analysis was carried out by means of STATISTICA 10.0 Pl. The non-parametric Wilcoxon signed rank test was used to assess the significance of the differences of the variables tested with the FPI-6 and the FMS test. The differences were considered as statistically significant if the level of test probability was lower than the assumed level of significance (p<0.05). A paired t-test power analysis of exercise influence determined that at least 20 subjects were required to obtain a power of 0.8 at a two-sided level of 0.05 with effect size d = 0.8. This analysis was based on data derived from previous literature [17–19].

## Results

There were no statistically significant differences (p>0.05) in the values of the FPI-6 and the FMS test between Group 1 and Group 2 at baseline prior to the start of the training.

### The Foot Posture Index (FPI-6)

After 6 weeks of plantar short foot muscles exercising a significant improvement was observed in the following parameters: talar head palpation in Group 1, and inversion/eversion of the calcaneus in Group 2. Talar head palpation underwent a statistically significant change (p = 0.001) in Group 1 –there was a slight improvement of the foot posture from a slight foot pronation towards a neutral foot in both lower extremities. In Group 2 the change was not significant (p = 0.093) (Table 1). There was a statistically significant improvement (p = 0.017) in inversion/eversion of the calcaneus in Group 2 from a slight foot pronation towards a neutral foot in both lower extremities. In Group 1 there were no statistically significant differences (p = 0.384) (Table 1). There were no statistically significant changes in other parameters evaluated by the Foot Posture Index (Table 1).

**Table 1:** The FPI-6 test at baseline and after 6 weeks of exercising.

Test	Median		Min		Max		Variance		p
	B	P	B	P	B	P	B	P	
Talar head palpation									
Gr 1	1	0	0	0	1	1	0.25	0.13	**0.001**
Gr 2	1	1	0	0	2	1	0.42	0.14	0.093
Supra and infra lateral malleolar curvature									
Gr 1	0	0	0	0	2	1	0.55	0.22	0.141
Gr 2	1	1	0	0	2	2	0.56	0.41	0.796
Inversion/eversion of the calcaneus									
Gr 1	0	0	-1	-1	1	1	0.32	0,19	0.384
Gr 2	1	0	0	-1	1	1	0.25	0.08	**0.017**
Prominence in the region of the talonavicular joint									
Gr 1	1	0	-1	-1	1	1	0.64	0.28	0.348
Gr 2	1	0	-1	0	2	1	0.95	0.24	0.627
Height and congruence of the medial longitudinal arch									
Gr 1	0	0	-1	-1	2	1	0.91	0.34	0.318
Gr 2	0	0	-1	0	1	1	0.49	0.21	0.657
Abduction/adduction of the forefoot on the rear foot									
Gr 1	1	1	-1	0	2	2	0.69	0.68	0.070
Gr 2	1	1	0	0	2	2	0.60	0.56	0.380
Total score									
Gr 1	3	2	-2	-1	6	5	4.91	1.96	0.072
Gr 2	3	2	0	0	8	5	6.65	2.40	0.145

Gr—Group

B—Baseline

P—Post

p- p value

### The Functional Movement Screen (FMS)

There was a significant improvement in Group 1 in the movement patterns: deep squat, active straight leg raise and in total score. The improvement of deep squat performance was observed in both groups, but the change was statistically significant (p = 0.007) only in Group 1 (Table 2). The trend for improvement in active straight leg raise was observed in both groups. However, this parameter underwent a statistically significant change (p = 0.001) only in Group 1 (Table 2). The greater change in total score was observed in Group 1 (from 13 to 17 points) and it was statistically significant (p = 0.002). The improvement in Group 2 was also noted from 15 to 17 points, but that difference wasn’t statistically significant (p = 0.063) (Table 2). In other parameters there were no statistically significant changes (Table 2).

**Table 2:** The Functional Movement Screen test at baseline and after 6 weeks of exercising.

Test	Median		Min		Max		Variance		p
	B	P	B	P	B	P	B	P	
Deep squat									
Gr 1	2	2	1	2	2	3	0.23	0.30	**0.007**
Gr 2	2	2	1	1	2	2	0.33	0.15	0.083
Hurdle step									
Gr 1	2	2	1	2	3	3	0.24	0.26	0.054
Gr 2	2	2	1	1	3	3	0.38	0.44	0.166
In-line lunge									
Gr 1	2	2	1	1	3	3	0.57	0.42	0.053
Gr 2	3	3	1	1	3	3	0.60	0.38	0.338
Shoulder mobility									
Gr 1	3	3	1	1	3	3	0.60	0.35	0.337
Gr 2	3	3	2	2	3	3	0.15	0.08	0,338
Active straight leg raise									
Gr 1	2	3	1	2	3	3	0.57	0.19	**0.001**
Gr 2	2	2	1	2	3	3	0.56	0.20	0.062
Trunk stability push up									
Gr 1	2	3	1	1	3	3	0.41	0.73	0.273
Gr 2	2	2	1	1	3	3	0.81	0.69	0.081
Rotary stability									
Gr 1	2	2	1	2	2	2	0.19	0.00	0.082
Gr 2	2	2	1	1	2	2	0.27	0.20	0.081
Total score									
Gr 1	13	17	10	14	19	20	7.33	3.47	**0.002**
Gr 2	15	17	11	13	19	20	6.20	4.08	0.063

Gr—Group

B—Baseline

P—Post

p- p value

## Discussion

The most novel finding of this study is that the plantar short foot muscles exercises significantly modify the foot posture and reduce tendency to pronation in long distance runners. Moreover, these exercises have beneficial effect on fundamental movement patterns. Our results showed that both plantar short foot muscles training programs are effective, with mild superiority of Group 1 exercise program (“Vele’s Forward Lean” and “Reverse Tandem Gait”). Those effects and consequences may be significant in athletic training, and we will discuss them in further detail below. To date there is a lack of studies describing the use of similar exercises in long-distance runners. However, there are some studies, which are indirectly related to this subject.

We observed the change of the foot posture towards the neutral foot, after 6 weeks of plantar short foot muscles training programs in both groups. The significant change was noted in talar head palpation in Group 1 and inversion/eversion of the calcaneus in Group 2. There are some studies that describe the relationship between excessive foot pronation and increased risk of acute injury or repetitive strain injury [2,4,5]. Kernozek et al. [2] have reported that individuals with flat-arched foot pronated significantly more in stance than high-arched individuals. According to Cowley et al.[5] the changes in foot posture towards a more pronated position may have implications for foot function, and therefore risk of injury. It was also reported that excessive tibial internal rotation coupling with rearfoot eversion during the first half stance phase of running was associated with patella-femoral pain syndrome, Achilles tendon pain and shin splint [27]. Eslami et al.[28] have reported high correlation (r = 0.99) between rearfoot eversion and tibial internal rotation during the first 50% stance phase of gait.

Lynn et al. [17] compared the effects of the plantar intrinsic foot muscles exercises: the short foot exercise and the towel curl exercise. The second exercise involves a contribution from the extrinsic muscles, such as the flexor digitorum longus. During the dynamic balance test in both groups they observed a decrease in the center of pressure deviations range in the mediolateral direction. However, the improvement was more significant in group with the short foot exercise.

Similar results were reported by Drewes et al. [29]. They evaluated the effectiveness of the short foot exercises in patients with lower extremity injuries. Four-week therapy has improved the dynamic parameters: test of the intrinsic foot muscles and step down test. There were no differences in the static measures, assessment of the calcaneus. However, in our study, there was a statistically significant improvement of calcaneal frontal plane position from a slight foot pronation towards a neutral foot in both lower extremities.

Mulligan and Cook [19] evaluated the influence of four-week intrinsic short foot exercise on the medial longitudinal arch morphology and dynamic function. They noted lower values in the navicular drop test, indicating a smaller range of foot pronation. The data from our study confirm these results.

There are some reports describing the use of the Foot Posture Index in athletes in the literature to date. Cowley and Marsden [5] evaluated the change of FPI-6 and navicular height in runners after the half marathon. The researchers noted the significantly decreased height of the navicular bone in the both feet, indicating the higher degree of foot pronation. However, that change was significant only in the left foot, the total score of the Foot Posture Index increased by 1,7. The change was not statistically significant in the right foot—the value increased only by 0,3. Those results confirm the necessity of applying the exercises which activate the plantar short foot muscles and enhance the medial longitudinal arch in runners. In the mentioned study, the baseline FPI-6 value was +3 for both feet which allowed the researchers to classify the foot into the neutral foot, with a slight tendency towards pronation, and which is in agreement with FPI-6 baseline values from our study (+3 for the left foot and +2 in the right foot).

Similar study was conducted by Escamilla-Martinez et al.[6], who evaluated the foot posture using the Foot Posture Index and plantar pressure distribution in 30 runners before and after a run at a moderate pace (3.3 m/sec), continued for 60 minutes. They observed the tendency towards foot pronation. The total score in FPI-6 increased by 2 points in both feet, the pressure under the medial heel and the second metatarsal head increased, and the longitudinal arch decreased.

In our study after 6 weeks of plantar short foot muscles exercising the significant improvement was observed in deep squat and active straight leg raise. The changes occurred in both groups, but were statistically significant only in Group 1. These results demonstrated that the applied therapeutic programs may reduce susceptibility to injuries by improvement in fundamental movement patterns.

The primary goal of the Reverse Tandem Gait exercise is to facilitate intrinsic short foot muscles and to control posterior chain muscles. Walking backward (Reverse Tandem Gait) is an open chain exercise, where the foot is properly, axially loaded, and the subject learns the appropriate pattern of load transfer from toes to heel [25,26]. Appropriate motor control of load transfer from toes to heel is the necessary condition of a proper squat performance. Therefore, Reverse Tandem Gait in addition to the short foot muscles facilitation, also positively affects the proximal body segments, improving the hip mobility, the neuromuscular control of hip movement in relation to pelvis, and control of load transfer from toes to heel [25,26,30]. All of those elements are necessary for a proper performance of the active straight leg rise test and the deep squat test [25,30]. All of the elements may contribute to the improvement in FMS test observed in our study.

Both sets of exercises performed by the runners in our study activate the intrinsic short foot muscles and favor the proper feet loading. In both groups, we noted the improvement in the active straight leg rise and the deep squat tests, but the change was significant only in Group 1, in which the Reverse Tandem Gait was practiced. We hypothesized that the difference between groups may also occur because of a wider impact of Reverse Tandem Gait than Janda’s short foot exercise on musculoskeletal system, and especially on the superficial back line muscles [9,26]. Some authors have observed that exercise focusing on plantar foot muscles may increase the flexibility and range of motion proximally in lower limb and in lumbar spine [12,31,32]. They suggested that this effect may be achieved due to anatomical and functional connections between fascia and muscles in the superficial back line [31,32]. That may also be a one of the reasons why we observed the improvement in fundamental movement patterns after 6 weeks of performing short foot muscles strengthening exercises. The reciprocal tension transfer from foot muscles to proximal parts of the superficial back line and from upper parts to foot and resulted clinical implications have been reported previously [13–15]. Some studies suggested that tightness of the posterior muscles of the lower limb could be involved in plantar fasciitis etiology [13–15]. Moreover, the risk factors of plantar fasciitis are sport or physical exercises [33], increased subtalar pronation accompanying pes cavus or flat feet and limited ankle dorsiflexion [14]. Therefore, runners who have weak short foot muscles resulting in excessive foot pronation may be in high risk of plantar fasciitis. The results of some studies [12,13] have indicated that the functional deficit of the plantar fascia is also related to hamstring shortening and restricted range of motion in the ankle. Restricted foot dorsiflexion may be compensated by excessive pronation of the subtalar joint and causing tension on the plantar fascia [14]. Therefore, we have suggested that short foot muscles strengthening exercise may have beneficial effect in prevention of excessive foot pronation and plantar fascia overload and may decrease the risk of injuries in long distance runners.

There are several limitations of this study that need to be addressed. First, the study population consisted of healthy subjects in which the baseline measurement classified the foot into the neutral foot using the Foot Posture Index, so these findings may not be able to be extrapolated to a population with severe foot pronation and future research should be conducted with such runners. Also it should be noted that subjects in this study ran 3–7 times a week with a total distance of 30–100 km per week which may have influenced the group homogeneity. Lack of control group is also a study limitation. Comparison of two kinds of exercises allow us to observe the difference between homogenous gropus of runners, but the inclusion of non exercising group to the study protocol would be beneficial. Also it should be noted that FPI-6 allow us to evaluate the foot posture only in static condition, therefore the results may not be fully extrapolated to a dynamic condition.

## Conclusions

The observed change of foot posture from a slight pronation towards a neutral foot evaluated by the Foot Posture Index suggests that the applied exercises may have beneficial effect on foot alignment in long distance runners. The higher total score in the Functional Movement Screen test suggests, that the applied exercises may improve the quality of the fundamental movement patterns, and thus may reduce the risk of injury. Based on our results, we may suggest that both training programs are effective with slight superiority of Vele’s Forward Lean and Reverse Tandem Gait.

## Practical Implications

The plantar short foot muscles exercises significantly modify the foot posture and reduce tendency to pronation in long distance runners. The plantar short foot muscles exercises may improve the quality of the fundamental movement patterns, and thus, may reduce the risk of injury. An optimal training of the plantar short foot muscles in long-distance runners should be incorporated in daily training routine.

## Supporting Information

S1 Checklist Trend statement checklist. (PDF)

S1 Protocol Bioethical protocol of this study—English. (PDF)

S2 Protocol Bioethical protocol of this study—Polish. (PDF)
