# Supplementary material for: The Influence of Plantar Short Foot Muscle Exercises on Foot Posture and Fundamental Movement Patterns in Long-Distance Runners, a Non-Randomized, Non-Blinded Clinical Trial
Source: PLoS One. 2016 Jun 23;11(6):e0157917. doi: 10.1371/journal.pone.0157917 (PMC4918976; doi:10.1371/journal.pone.0157917)
Supplement: S1 Protocol — (PDF) [file pone.0157917.s002.pdf]

The Ethical Committee of Regional Medical Chamber in Krakow
ul. Krupnicza 11a,
31-123 Kraków, Poland
biuro@oilkrakow.org.pl
Tel: (4812) 6191720
Faks: (4812) 6191730

The evaluation the efficacy of functional training based on „joint-by-joint" conception in long distance runners.

Running is one of the most common forms of movement activities but, unfortunately, dangers of injuries or strains are high on both professional and amateur level. When an injury takes place it triggers sequence of changes in movement apparatus causing consequential dysfunctions. Whereas strains cause adoption of abnormal movement patterns which cause whole body functioning limitations.

Risk factors of foot injuries constitute a whole separate group especially in runners. It comprises dysfunction of biomechanics or anatomical structure of foot, decreased flexibility, muscular misbalance or weakened short muscles in the plantar side of the sole, structure of bedding and type of running shoes. Excessive pronation is often considered to be an injury causing factor.

Conception of functional training developed as a reaction to the phenomenon of increased number of injuries and strains caused by the rise of interest in various types of physical activities in general public. Functional training is understood as a specific set of exercises aimed to establish essential movement patterns vital for normal development of motor abilities in athletes. It ensures separate elements of bio-kinematic chain are combined into an optimal movement pattern. Functional training is employed to improve physical fitness and results in sports, and to decrease injury risks.

American physiotherapist Gray Cook is considered to be one of the forerunners of functional training. He was the author of Functional Movement Screen (FMS) – an examination which allows to analyze essential movement patterns and detect personal limitations, asymmetries or dysfunctions which could potentially cause an injury.

Main assumption of "joint-by-joint" therapy conception is a holistic approach to an athlete or a patient. Insufficient stability in a certain body area (e.g. lumbar spine) could be caused by insufficient mobility in the area nearby (e.g. limited hip-joint extension). If there is unsufficient mobility in one area it will be compensated for in the other one. Thus therapy should not be focused on the problem area, instead a comprehensive analysis of the entire kinematic chain as well as all basic movement patterns should be performed. According to "joint-by-joint" conception therapy should begin first with exercises improving mobility, from the ankle up, segment by segment, then continue with improving stability.

Exercises which activate inner foot muscles on the plantar side aim to restore strength and normal muscle tension as well as strengthen medial longitudinal arch, what, in its turn, increase stability in the ankle. Those exercises are employed both in sports and rehabilitation. They are advised in cases of ankle injuries, pes planus, claw and hammer toes as well as an element of sensomotor training. But have not found any reports on employment of those exercises in runners.

Functional examination, by means of detection of all limitations and asymmetries, allows to employ individually customized trainings, which could stimulate restoration of correct movement patterns and decrease injury risks in athletes and patients.

Aims

The aim of the presented research is to define influence of exercising short plantar foot muscles on foot placement and functional movement patterns in runners. Above that we had the following objective: to assess efficiency of functional training based on “joint-by-joint” therapy conception, customized to compensate individual limitations and dysfunctions in runners. Interactions between functional training and quality of essential movement patterns, generated lower limbs power and distribution of load on feet in statics and dynamics were considered.

Material and methods

Study group

The research covered 60 amateur regular long distance runners aged 20-45 years old.

Methods

We used the following scientific techniques and tools:

- Survey – by means of questionnaire on training, state of health, history of injuries and possible rehabilitation as a consequence.
- Functional Movement Screen (FMS) - a comprehensive examination performed to analyze essential movement patterns. The aim of FMS is to any detect personal limitations, asymmetries or dysfunctions which could potentially cause an injury. It consists of seven movement tasks performance of which requires usage of stability, mobility, strength and coordination of employed muscle groups combined, as well as normal nervous muscular control.
- Baropodometry platform-used to measure power distribution of feet on the floor. It is used for examining posture control power of individuals standing still, walking or running. Also it is able of analyzing the way individuals maintain their balance.
- plyometrics platform-allows to assess plyometrics, power and explosiveness of lower limbs during jumps.

- Dynamometric examination of lower limbs joints- used to measure lower limbs strength concentrically and eccentrically. It provides data on maximum torque, value of angle, total work and average power.
- The Foot Posture Index (FPI-6)[19] is used for multidimensional and comprehensive evaluation of the feet. Due to its non-specific character, it has a wide clinical application in assessment of the risk of injury in athletes

Functional training employed by us consists of two stages. The first one lasts 6 weeks and is devoted to activation of inner foot muscles on the plantar side. The second stage lasts also 6 weeks and is devoted functional training, which comprises exercises individually customized for each runner aiming to correct their particular dysfunctions. This functional training aims to improve mobility and stability.

Each subject will be examined three times: before the first stage, after the first stage and after the second stage. Trainings will be done every day, besides one training a week will be done under the therapist control.
