# Supplementary material for: The Influence of Plantar Short Foot Muscle Exercises on Foot Posture and Fundamental Movement Patterns in Long-Distance Runners, a Non-Randomized, Non-Blinded Clinical Trial
Source: PLoS One. 2016 Jun 23;11(6):e0157917. doi: 10.1371/journal.pone.0157917 (PMC4918976; doi:10.1371/journal.pone.0157917)
Supplement: S2 Protocol — (PDF) [file pone.0157917.s003.pdf]

Komisja Bioetyczna przy Okręgowej Izbie Lekarskiej w Krakowie
ul. Krupnicza 11a
31-123 Kraków, Polska
biuro@oilkrakow.org.pl
Tel: (4812) 6191720
Faks: (4812) 6191730

Ocena skuteczności treningu funkcjonalnego w oparciu o koncepcję „joint-by-joint” u biegaczy długodystansowych.

Bieganie jest jedną z najbardziej rozpowszechnionych form aktywności ruchowej. Niesie jednak ze sobą ryzyko wystąpienia urazów lub przeciążeń, zarówno na poziomie wyczynowym, jak i amatorskim. Przebyty uraz wywołuje kaskadę zmian w układzie ruchu, doprowadzając w konsekwencji do powstania dysfunkcji. Natomiast w sytuacji zmian przeciążeniowych dochodzi do powtarzania nieprawidłowych wzorców ruchowych, które zaburzają funkcjonowanie całego organizmu i prowadzą do ograniczeń.

Wśród wielu czynników ryzyka wystąpienia urazów u biegaczy, jedną grupę stanowią te związane ze stopą. Obejmują one zaburzenia biomechaniki lub budowy anatomicznej stopy, zmniejszoną elastyczność, dysbalans mięśniowy lub osłabioną siłę mięśni krótkich po stronie podeszwowej, rodzaj podłoża oraz typ obuwia. Nadmierna pronacja często jest zaliczana do czynników przyczyniających się do wystąpienia kontuzji.

Jako odpowiedź na coraz częstsze pojawianie się urazów i przeciążeń, wynikających ze wzrostu zainteresowania różnymi formami aktywności, zrodziła się koncepcja treningu funkcjonalnego. Jest on rozumiany jako specyficzny zestaw ćwiczeń, ukierunkowany na kształtowanie podstawowych wzorców ruchowych, stanowiących fundament prawidłowego rozwoju zdolności motorycznych zawodnika. Łączy w sobie prawidłowe działanie poszczególnych elementów łańcucha biokinematycznego w optymalny wzorzec ruchowy. Celem jego stosowania jest poprawa poziomu przygotowania motorycznego oraz wyniku sportowego, a także obniżenie ryzyka urazu.

Jednym z prekursorów treningu funkcjonalnego był amerykański fizjoterapeuta Gray Cook. Jest on autorem kompleksowego badania – Functional Movement Screen (FMS) – pozwalającego ocenić jakość podstawowych wzorców ruchowych oraz umożliwiającego wykrycie indywidualnych ograniczeń, asymetrii lub dysfunkcji, które potencjalnie mogą doprowadzić do wystąpienia urazu.

Założeniem terapii opartej na koncepcji „joint-by-joint" jest całościowe spojrzenie na zawodnika lub pacjenta. Brak stabilności w miejscu, gdzie byłaby ona pożądana (np. w odcinku lędźwiowym kręgosłupa) może wynikać z braku mobilności w sąsiednim obszarze (np. ograniczenie wyprostu w stawie biodrowym). Gdy brakuje ruchu w jednym odcinku, zostanie on kompensacyjnie wykonany w innym miejscu. Dlatego więc nie należy skupiać się jedynie na obszarze występowania dolegliwości, ale dokonać kompleksowej oceny całego łańcucha kinematycznego i wszystkich podstawowych wzorców ruchowych, a następnie przeprowadzić terapię. Według koncepcji „join-by-joint" należy rozpocząć od ćwiczeń poprawiających mobilność, idąc segmentami od stawu skokowego w górę, a następnie stabilność.

Ćwiczenia aktywizujące wewnętrzne mięśnie stopy po stronie podeszwowej przywracają siłę i prawidłowe napięcie mięśniowe oraz wzmacniają przyśrodkowy łuk podłużny stopy, co wpływa na zwiększenie stabilności stawu skokowego. Ćwiczenia te mają zastosowanie w sporcie i rehabilitacji. Są zalecane między innymi po urazach stawu skokowego, w płaskostopiu, w deformacjach takich jak palce szponiaste i młoteczkowate, oraz jako element treningu sensomotorycznym. W literaturze przedmiotu brak jest doniesień na temat wykorzystania powyższych ćwiczeń u biegaczy.

Badanie funkcjonalne poprzez identyfikację wszelkich ograniczeń i asymetrii, umożliwia wprowadzenie odpowiedniego, indywidualnie dobranego treningu. Może on przyczynić się do przywrócenia prawidłowych wzorców ruchowych oraz do zmniejszenia ryzyka urazu u sportowca lub pacjenta.

Cel badań

Celem niniejszej pracy będzie zbadanie wpływu ćwiczeń krótkich mięśni strony podeszwowej stóp na postawę stopy oraz na funkcjonalne wzorce ruchowe u biegaczy. Ponadto celem badań będzie ocena skuteczności treningu funkcjonalnego, opartego o koncepcję „joint-by

joint”, ukierunkowanego na indywidualne ograniczenia i dysfunkcje u biegaczy, ze szczególnym uwzględnieniem jego wpływu na jakość podstawowych wzorców ruchowych, wynik generowanej mocy kończyn dolnych, rozkład obciążeń stóp w statyce i dynamice.

Materiał i metody

Grupa badana

Grupę badaną stanowić będzie 60 osób regularnie trenujących biegi długodystansowe na poziomie amatorskim, w wieku od 20 do 45 lat. Osoby badane będą rekrutowane z klubów biegowych zrzeszających biegaczy amatorów działających na terenie miasta Krakowa. Do badań zakwalifikowane będą osoby biegające z częstotliwością od trzech do siedmiu razy w tygodniu, pokonujące od 30 do 110 kilometrów tygodniowo . Z badań wykluczone zostaną osoby, które w okresie 6 miesięcy poprzedzających badania przebyły ostry uraz lub ostry stan przeciążeniowy narządu ruchu. Zastosowany u biegaczy trening funkcjonalny będzie składał się z dwóch etapów.

W pierwszym z nich (trwającym 6 tygodni), wykonywane będą ćwiczenia angażujące krótkie mięśnie strony podeszwowej stóp. W drugim etapie, trwającym również 6 tygodni, zostanie wprowadzony trening funkcjonalny, zawierający ćwiczenia dostosowane indywidualnie dla każdego biegacza, nakierowane na zaobserwowane u nich dysfunkcje. Celem tego treningu będzie poprawa mobilności i stabilności.

Metody

Zastosowane zostaną następujące techniki i narzędzia badawcze:

- Wywiad – jako narzędzie posłuży kwestionariusz wywiadu dotyczący treningu, stanu zdrowia, przebytych urazów i przeciążeń oraz stosowanej ewentualnej rehabilitacji.
- Test Functional Movement Screen (FMS) – jest to kompleksowe badanie, pozwalające ocenić jakość podstawowych wzorców ruchowych. Jego celem jest wykrycie indywidualnych ograniczeń, asymetrii lub dysfunkcji, które mogłyby doprowadzić do kontuzji. Składa się z siedmiu zadań ruchowych, których wykonanie wymaga połączenia stabilności, mobilności, siły i koordynacji zaangażowanych grup mięśniowych, oraz prawidłowej kontroli nerwowo-mięśniowej.

- Testy funkcjonalne dotyczące głównych grup mięśniowych – są to testy oceniające elastyczność poszczególnych mięśni. Badane będą między innymi mięśnie: czworogłowy uda, biodrowo-lędźwiowy, napinacz powięzi szerokiej uda, mięśnie pośladkowe, czworoboczny lędźwi, trójgłowy łydki. Każdy mięsień będzie badany w określonej pozycji wyjściowej.
- Platforma baropodometryczna - służy do pomiaru rozkładu sił nacisku stóp na podłoże. To urządzenie, które analizuje, którą częścią stopy badany mocniej naciska na płaszczyznę podparcia podczas stania, chodu i biegu. Pozwala również na badanie równowagi.
- Platforma do wyskoku – pozwala na ocenę skoczności, mocy oraz parametrów eksplozywnych kończyn dolnych podczas wyskoku.
-Badanie dynamometryczne stawów kończyn dolnych – służy do pomiaru siły mięśni

w kończyn dolnych w warunkach pracy koncentrycznej i ekscentrycznej. Pozwala na ocenę między innymi takich parametrów jak maksymalny moment obrotowy, wartość kątowa, praca całkowita, moc średnia.

Każdy uczestnik zostanie zbadany trzykrotnie: na początku badań, po zakończeniu pierwszego etapu oraz po zakończeniu treningu funkcjonalnego w drugim etapie. Trening będzie wykonywany codziennie, w tym 1 raz w tygodniu pod kontrolą fizjoterapeuty.

Opracowanie statystyczne wykonane zostanie przy użyciu programu STATISTICA 10.0 Pl. Dla oceny istotności różnic badanych zmiennych zastosowano test ANOVA oraz test Wilcoxona. Różnice zostaną uznane za istotnie statystycznie, jeśli poziom prawdopodobieństwa testowego będzie niższy od założonego poziomu istotności ($p< 0,05$).
